# An open label pilot study of micro expression recognition training as an intervention for low mood

**DOI:** 10.1038/s41598-025-01132-w

**Published:** 2025-05-19

**Authors:** Kasia Wezowski, Ian S. Penton-Voak

**Affiliations:** 1 School of Psychological Science, University of Bristol, Bristol, UK; 2 National Institute for Health Research Bristol Biomedical Research Centre, University Hospitals Bristol NHS Foundation Trust, Bristol, UK

**Keywords:** Low mood, Micro expressions, Depression, Training, Social functioning, Psychology, Human behaviour

## Abstract

The present study is a pilot, open-label study which assessed whether training using the Micro Expression Training Tool for 3 weeks is effective in improving micro expression processing, mood, and social functioning. Participants completed four training sessions and could practice additionally, if they wished to. Measures of low mood and social functioning were made prior to and after the training in order to assess potential therapeutic effects. The results showed strong evidence for an effect of training on improvement in micro expression recognition, reflected in higher scores after training, and an association between the number of sessions done and the degree of improvement. In an adjusted model, there was an improvement in low mood, which should be investigated in future studies. There was no effect of micro expression recognition training on social functioning. This pilot study demonstrates a new, effective method for training micro expression processing and shows that it has potential for mood improvement, which may be beneficial for the halting of sub-clinical depression.

## Introduction

Micro expressions are facial expressions of seven emotions, expressed in different cultures around the world^1^—anger, contempt, disgust, fear, happiness, sadness, and surprise^2^ elicited spontaneously^3^ in less than half a second^4,5^. Emotional facial expressions play an important role in communication and are used as information about how the other person is feeling^6^. Micro expressions are involuntary and difficult to hide, and thus appear even when we are trying to deceive others^1^.

Previous research has demonstrated that facial expression recognition, including recognition of micro expressions, is connected to one’s well-being. People experiencing depression show poorer overall performance on facial expression recognition tests^7–11^. Also, depressed people show a negative bias in emotion recognition, seeing, for instance, happy faces as neutral and neutral faces as sad^10,11^. This has also been demonstrated with micro facial expressions^12^—people with more depressive symptoms are overall worse at micro expression recognition, as well as at happy faces recognition. Furthermore, higher depression scores are linked to a bias towards perceiving neutral faces as sad.

Emotion recognition is a part of social signalling, which is a vital aspect of our daily lives that is commonly impaired in mental health conditions^13^. Furthermore, it has been postulated that an important pathway for recovering from depression is through learning and applying new ways of interacting in social situations^14^. The neurocognitive model of depression posits that change towards more positive emotion processing at a certain time point predicts a larger improvement in symptoms of depression in the future^14^. In simpler terms, the more someone changes their emotion perception towards more positive assessment at time (1) the more their depression symptoms improve at time (2) indicating a causal relationship^14–16^. Hence, training people in the reduction of negative bias in emotion recognition is a promising and cost-effective idea for depression management^17^.

Many studies^18–25^ have indicated that (micro) facial expression recognition can be improved through training. No studies in the past have attempted to assess the effects of micro expression recognition training on depression. Furthermore, previous research has utilized tests such as the Micro Expression Training Tool^26^, which rely on static images in order to present emotions. Micro Expression Training Videos (METV) has the advantage of representing emotions through video, which makes it more similar to the way in which emotions are observed in real life, increasing its ecological validity. Furthermore, the METV training program includes teaching participants the muscle movements related to each micro expression, as well as letting them train on a large set of videos. Thus, it was expected that using METV for training will be effective in improving micro expression recognition. Furthermore, if the training leads to a reduction in cognitive bias towards perceiving stimuli as negative, there may also be a positive impact on mental health. The pathway of such an influence would be through ameliorating the cognitive bias present in depression. If the training does have a positive effect on depression symptoms, it could be a useful tool for the prevention of depression development in its earlier stages, a very important goal for clinical practice in the twenty-first century^27^.

The primary aim of this study was to examine the effect of micro expression recognition training (using Micro Expression Training Videos) on micro expression recognition in a sample of participants with low mood, and to evaluate whether such training has potential as a therapeutic intervention to improve low mood and social functioning. Based on previous literature, the following hypotheses were formulated:

### H1

*There will be an increase in micro expression recognition (METV scores) after participants complete the training.*

### H2

*There will be a decrease in depressive symptoms (Beck Depression Inventory—II—BDI-II scores) after participants complete the training.*

### H3

*There will be an increase in social functioning (Social Functioning Questionnaire—SFQ scores) after participants complete the training.*

## Results

### Sample characteristics

**Fig. 1:**
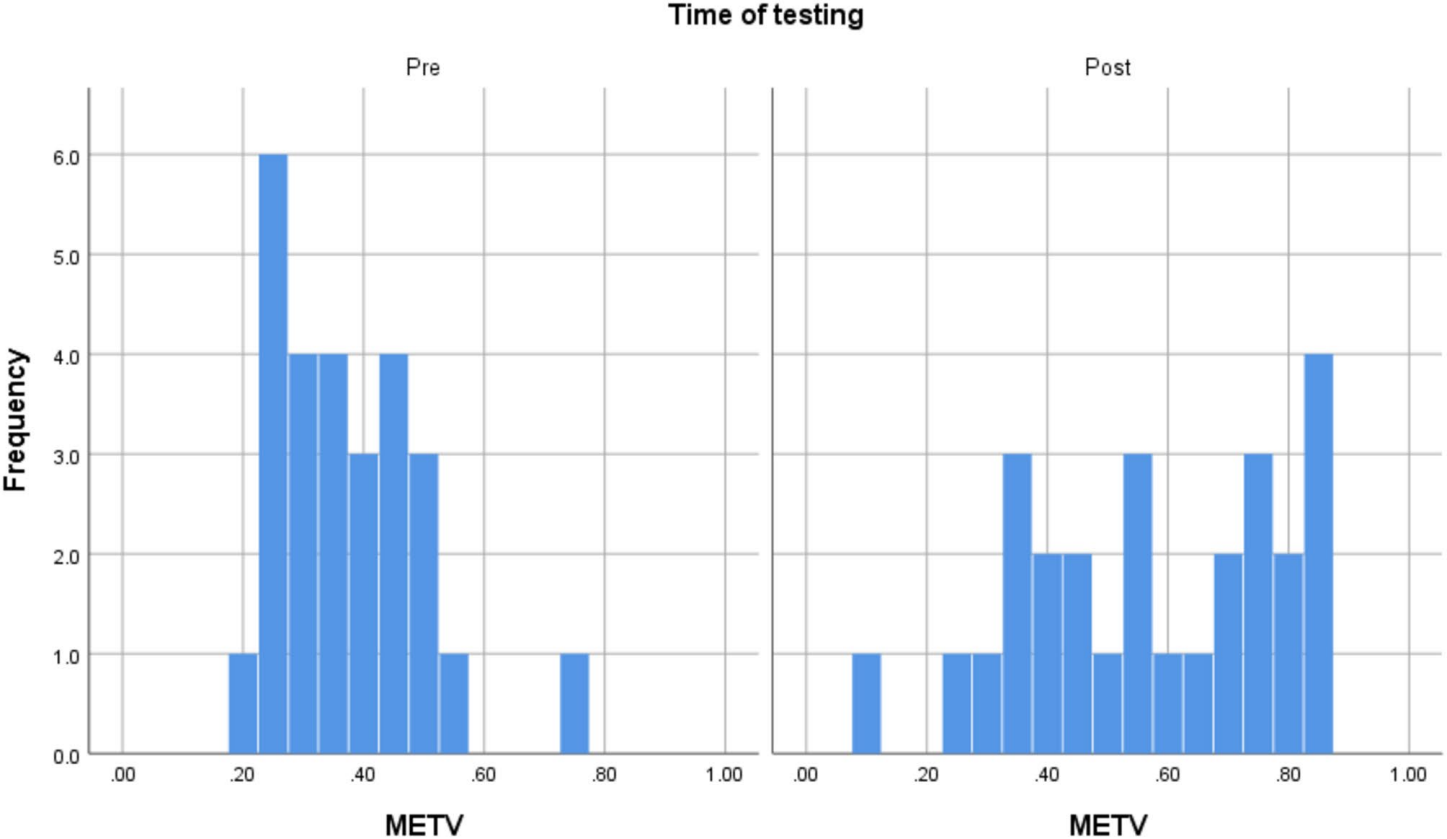
Histogram of METV distributions prior to and after training.

**Fig. 2:**
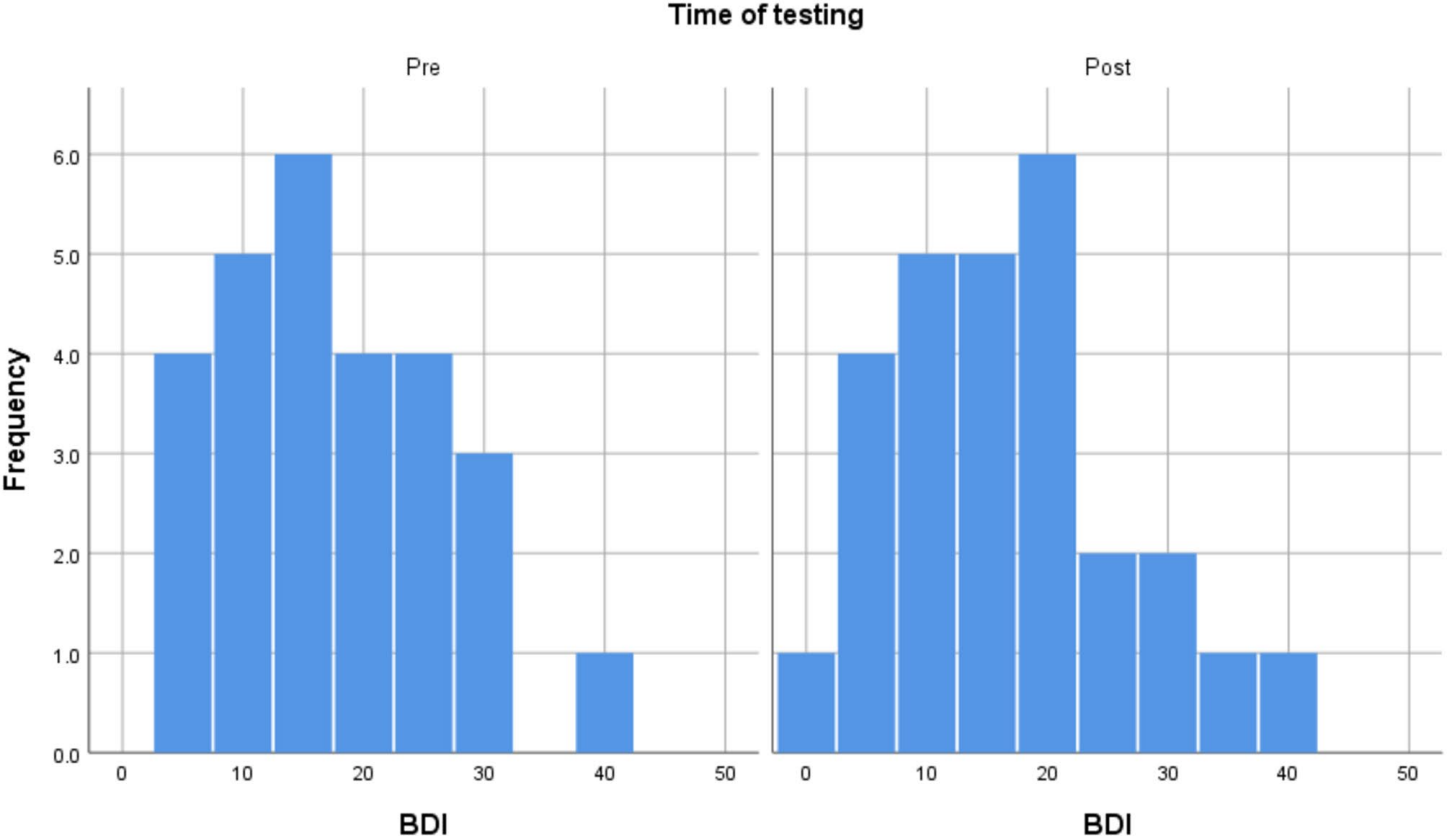
Histogram of BDI distributions prior to and after training.

**Fig. 3:**
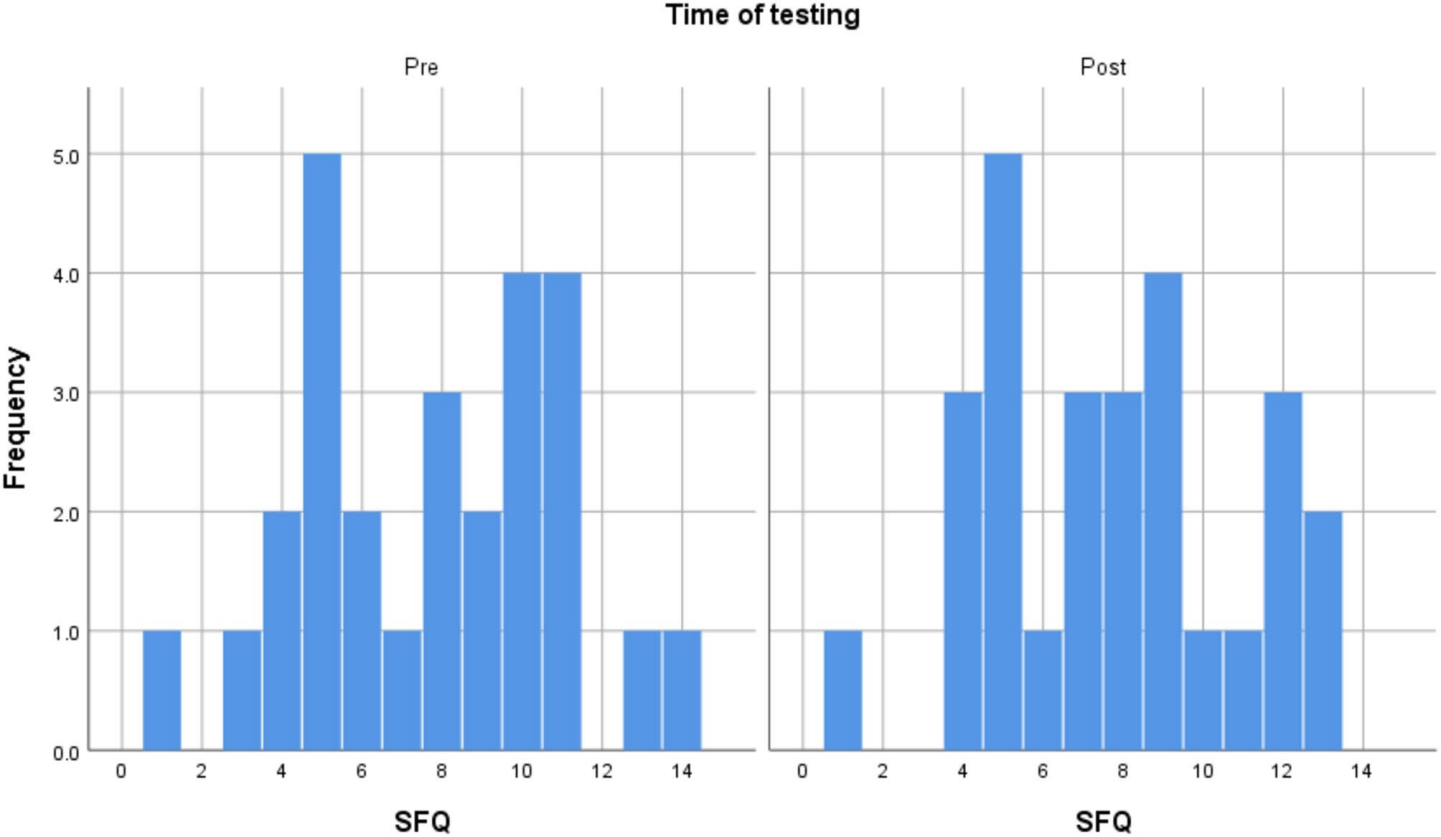
Histogram of SFQ distributions prior to and after training.

**Fig. 4:**
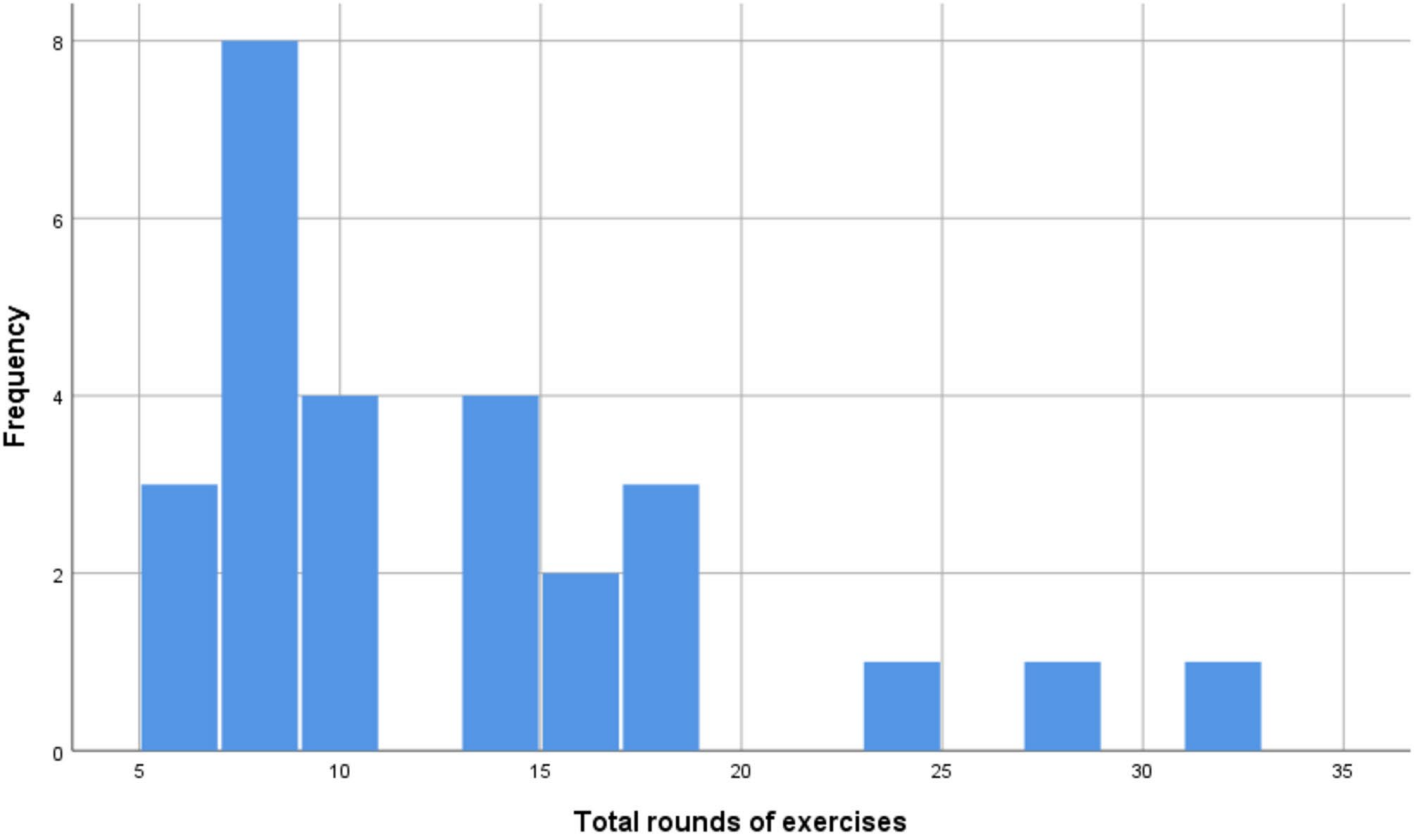
Histogram of the number of rounds of training done by participants.

Out of the 28 participants who took part in the study, one experienced significant technical difficulty and was removed from the study sample. Screening for outliers demonstrated that there were no extreme outliers. The final sample, thus, consisted of 27 participants (55.6% female). Their age was between 21 and 68, with an average of 40 (SD = 12.073). The participants were diverse in terms of education. 33% of the sample had an GCSE level education (11 years of education), 18.5% finished A level education (13 years of education), and another third had an undergraduate degree, and 14.8% had a postgraduate degree. As Table 1 demonstrates, they had a 37.4% success rate on METV on the first try in the first session and 57.2% in the last session. Their BDI and SFQ scores indicated mild levels of depression and mild difficulties in social functioning on average. The distributions of METV, BDI, and SFQ scores before and after the training may be observed in Figs. 1, 2, and 3, respectively. Adherence to training was good, with an average of 12.26 sessions done (SD = 6.67), and all participants doing at least 6 sessions. Around half of the sample (48.1%) did between 6 and 9 sessions, 40.8% did between 10 and 19, and finally, 11.1% did more than 20 (up to 32). A more detailed breakdown of the number of rounds of training done by the participants can be seen in the histogram in Fig. 4.

**Table 1:** Descriptive statistics of key study variables.

Variable	Mean	Std. Deviation	Minimum	Maximum
METV score pre training	0.37	0.12	0.2	0.75
METV score post training	0.57	0.21	0.1	0.85
BDI pre	17.70	9.06	4	39
BDI post	16.44	9.69	0	38
SFQ pre	7.74	3.24	1	14
SFQ post	7.70	3.17	1	13
Rounds of exercise	12.26	6.66	6	32

METV, Micro Expression Training Videos; BDI, Beck Depression Inventory-II; SFQ, Social Functioning Questionnaire.

### Hypothesis testing

**Fig. 5:**
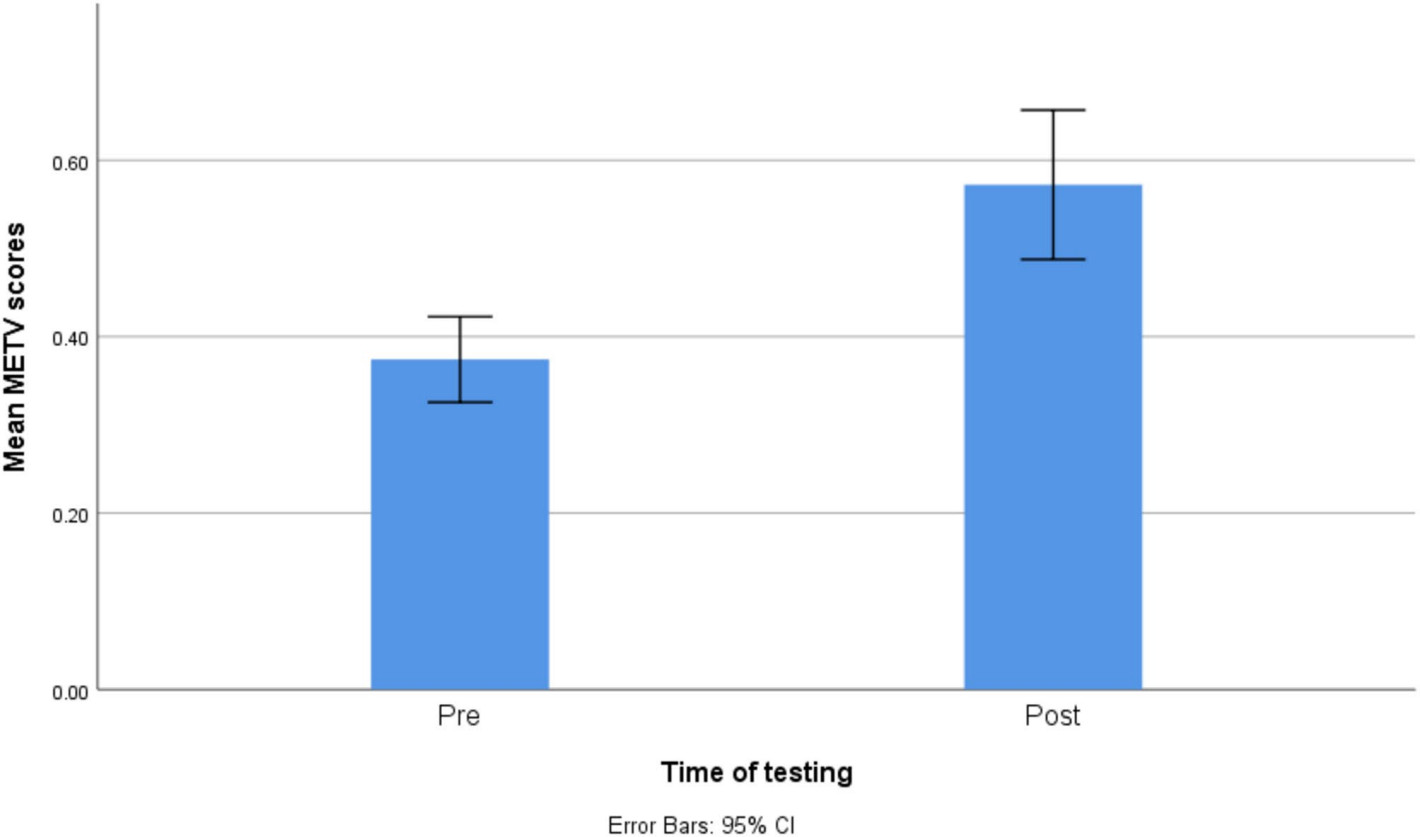
Bar chart demonstrating the difference in METV scores before and after the training.

In order to test the first hypothesis, three repeated-measures ANCOVAs were conducted with time of testing (pre vs post training) as the predictor (only predictor in first model) and age, gender, and education as covariates (in the adjusted model). There was strong evidence for a difference in METV scores before and after the training (Table 2), with a medium effect size (η^2^ = 0.45). The improvement in METV scores after training may also be seen in Fig. 5. However, there was no such evidence in the adjusted model. The third model, which was adjusted for the number of METV sessions done by a participant, demonstrated that it is the most important factor determining the degree to which the METV scores changed between the first and the second time of measurement, with a large effect size.

**Table 2:** ANCOVA models testing the change in METV scores pre and post training.

Predictor	Model 1			Model 2			Model 3		
	*F*	*p*	η^2^	*F*	*p*	η^2^	*F*	*p*	η^2^
Time of testing (pre vs post)	20.92	< 0.001	0.446	1.17	0.29	0.049	0.09	0.765	0.004
Gender				0.01	0.946	< 0.001	0.05	0.822	0.002
Age				0.77	0.389	0.032	1.53	0.230	0.065
Education				0.19	0.664	0.008	0.17	0.680	0.008
METV rounds of training							5.01	0.036	0.185

Model 1: unadjusted, Model 2: adjusted for gender, age, and education, Model 3: adjusted for gender, age, education, and number of rounds of training.

In order to test the second hypothesis, another set of ANCOVA tests were conducted with the same predictors as in the previous set of analyses, but BDI scores as the criterion (Table 3). In the unadjusted model, there was no evidence for a difference in BDI scores between the two times of testing. After adjusting the model, strong evidence for a reduction in the BDI scores was found, with a large effect size. While adjusting for age, gender, and education the BDI score dropped from 17.70 (95% CI 13.91, 21.49) to 16.44 (95% CI 12.52, 20.36).

**Table 3:** ANCOVA models testing the change in BDI scores pre and post training.

Predictor	Unadjusted			Adjusted		
	*F*	*p*	η^2^	*F*	*p*	η^2^
Time of testing (pre vs post)	1.80	0.191	0.065	10.32	0.004	0.310
Gender				0.01	0.959	< 0.001
Age				0.82	0.374	0.035
Education				0.11	0.743	0.005

Adjusted model adjusted for gender, age, and education.

In order to test the third hypothesis, a final set of ANCOVAs was conducted. The analyses (Table 4) showed that there was no evidence for effects of training, gender, age, or education on SFQ scores either in the unadjusted or the adjusted model.

**Table 4:** ANCOVA models testing the change in SFQ scores pre and post training.

Predictor	Unadjusted			Adjusted		
	*F*	*p*	η^2^	*F*	*p*	η^2^
Time of testing (pre vs post)	0.01	0.91	< 0.001	1.63	0.214	0.066
Gender				0.49	0.489	0.021
Age				0.07	0.79	0.003
Education				0.02	0.882	0.001

Adjusted model adjusted for gender, age, and education.

### Exploratory analyses

**Fig. 6:**
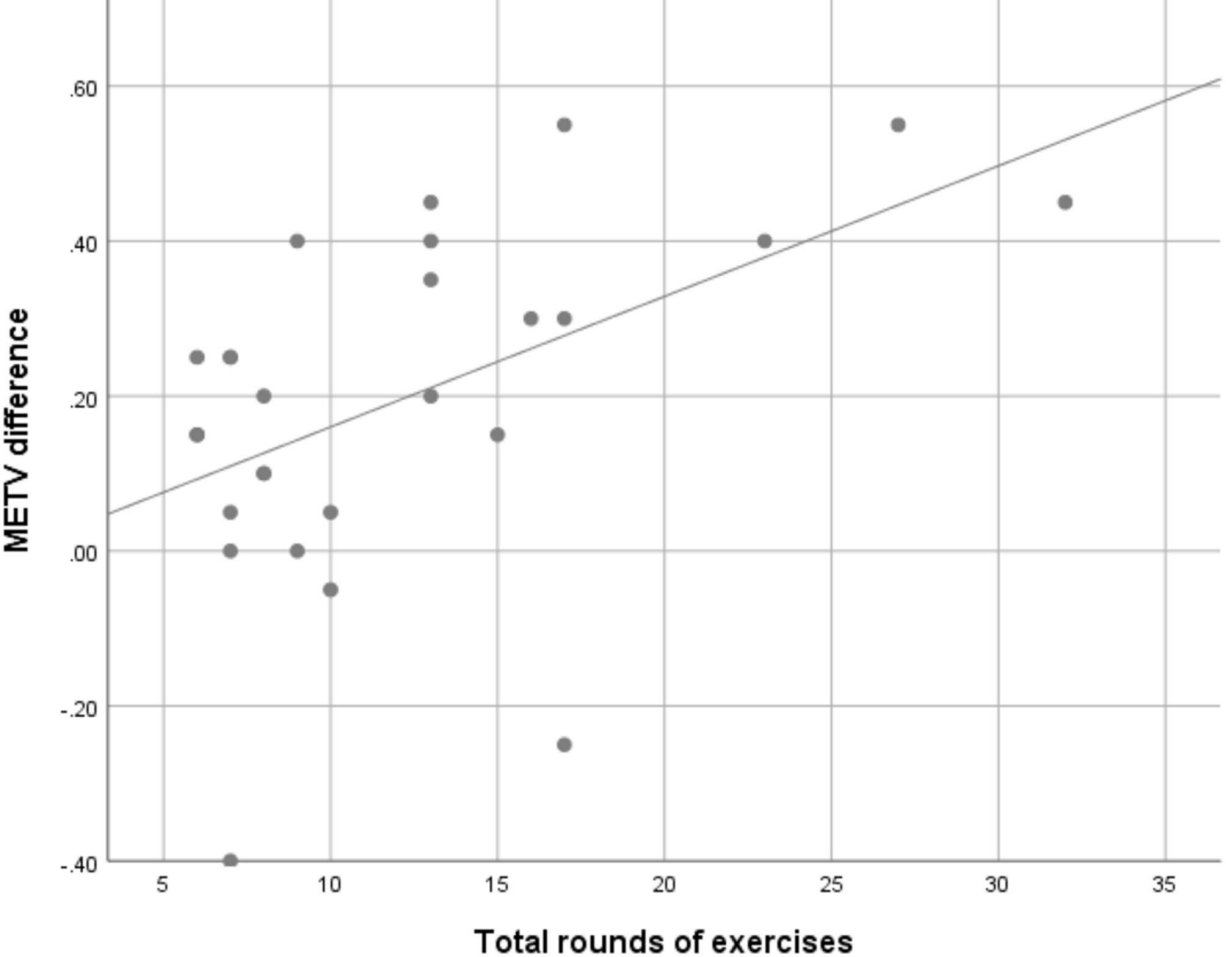
Scatterplot of the relationship between the rounds of exercises done by a participant and the change in their METV scores.

A series of ordinary least squares regressions (Table 5) was conducted in order to predict the factors that affected the change in the METV score. The first model showed evidence of a positive effect of rounds of exercise done on METV score change, *F* (1, 25) = 8.285, *p* = 0.008. The linear relationship between the two may be seen in Fig. 6. The second model showed weak evidence of an overall effect when adjusted for age, gender and education, *F* (4, 22) = 2.277, *p* = 0.093, and moderately strong evidence for the effect of rounds of exercise on the difference in METV scores. The reduced strength of evidence is a consequence of the increased number of predictors and the small sample size.

**Table 5:** Regression models predicting change in METV score pre and post training.

Predictor	Unadjusted		Adjusted	
	*B* [95% CI]	*p*	*B* [95% CI]	*p*
Rounds of exercise	0.017 [0.005, 0.029]	0.008	0.016 [0.004, 0.029]	0.017
Gender			− 0.059 [− 0.225, 0.108]	0.473
Age			0.001 [− 0.006, 0.008]	0.753
Education			− 0.030 [− 0.106, 0.046]	0.425

Adjusted model adjusted for gender, age, and education.

## Discussion

The present study was an exploratory investigation of the effects of three weeks of micro expression processing training on micro expression processing, low mood, and social functioning. Through testing of three hypotheses, the results showed that there was a relationship of micro expression processing training with micro expression processing and depressive symptoms, but not with social functioning. Additional analyses showed that the number of training sessions completed by a participant affected the degree of increase in their METV scores.

The first hypothesis expected an increase in METV performance after training. The participants showed improved performance after training, although the evidence for the difference was reduced after adjusting for age, education and gender. However, exploratory analyses indicated that the number of training sessions performed affected the difference in METV scores between the two time points, after adjusting for the demographic variables. This finding is in line with previous research demonstrating that it is possible to train facial expression recognition ability^18–25^. It also demonstrates that the effect of training using METV is stronger than that reported by other studies^19,20^, even with the small sample size. The fact that we were able to detect this effect in a pilot study with only 28 participants indicates that METV has promise in training people to improve their micro expression recognition. We also note that this relatively low sample size means adjustments for multiple demographic variables may lead to unstable results.

Due to the potential effects of emotion perception bias modification on depression^14,17^, we also tested whether or not the METV training affected low mood. It should be clarified that the present study uses the term “low mood” to refer to depression which is not clinically determined. Since the average participant in the present study scored somewhere between “mild mood disturbance” and “borderline clinical depression” on the BDI-II, and we used no other ways of determining whether they were clinically depressed, we opted to refrain from considering them as such. Finally, given the sampling done in the study was from the wide population and not from a clinical one (also clear from the fact that none of them were taking antidepressants), we considered it irresponsible to deem these individuals as depressed or not, given that they were not given a thorough clinical assessment. When testing in a non-adjusted model, there were no effects of training on depressive symptoms. However, after adjusting for age, gender and education, there was evidence for a reduction in low mood, with a moderate effect size. This is in line with previous findings indicating that there is a relationship between facial expression recognition and mental health indicators^8^, and shows that training using METV could be beneficial in improving one’s mental health. While the change in depression scores could have been a result of regression towards the mean^28^, these promising results demand further research which would test the results in a larger randomized controlled trial. If proved robust, the effect of micro expression training on mood improvement could be incorporated into depression prevention programs.

The results showed no effects of micro expression recognition training on social functioning, as measured by SFQ. There are two possible reasons for this—either the micro expression recognition training did not help improve social functioning, or the SFQ did not successfully measure this change, if it did occur. The time span of the study may have been too short to allow for the improvement in micro expression recognition to have an effect on social functioning of the participant.

The present study had several limitations. The first limitation was the small sample size, which was utilized due to the pilot nature of the study and the limited resources available. The second limitation was also a consequence of the small sample size and relates to a lack of a control group. Both of these limitations mean that the findings cannot be reliably generalized. However, the fact that an effect on improvement of micro expression recognition scores (in the unadjusted model) and on reduction in depressive symptoms (in the adjusted model) show promise for the potential of reliable effects to be found in a larger controlled study. Another limitation of the study is that no neurophysiological and/or biochemical assessments were done. Incorporating neurophysiological and biochemical assessments (e.g., fMRI, EEG, cortisol levels, inflammatory markers) could verify whether neural mechanisms of emotional processing are affected by METV, thus supporting the intervention for the potential it may have clinically. A final limitation is that there was no follow-up to determine how long lasting the effects of the intervention were. Again, since this was a pilot study, a future trial needs to be conducted in order to determine how long-lasting the effects of intervention are.

There were also significant strengths of the study. The design allowed for a simple intervention which could be used to teach people to recognize micro expression better through a fully automated, online process which can be finished in a few weeks, or even more quickly. The participants were able to do the training at a natural pace for them and as many times as they wished, and the effectiveness of the intervention on emotion judgements was further confirmed by the fact that those who did the training more also had better scores. Participants completed, on average, eight more sessions than minimum required for the study (four). These extra sessions were not financially compensated, suggesting that an intervention based on micro expression training would be acceptable to patients. Several participants also gave positive feedback to the study, noting that they enjoyed the training, which means that it would likely be relatively easy to motivate people to undergo the training in the future. Finally, the longitudinal design of the study made it possible for each participant to be their own control, rendering it improbable that the change in METV (or, to a lesser degree, BDI) scores to be a result of something other than the training that they went through.

## Conclusion

This study assessed the impact of a three-week micro expression training program on facial micro expression recognition, depression, and social functioning. The training was connected to enhanced micro expression recognition and reduced depression without affecting social functioning. The reduction in depression symptoms after training is consistent with the link between expression recognition and mental health, indicating potential benefits of METV training for mental well-being.

The study contributes to the facial recognition literature by demonstrating the efficaciousness of the METV, as the first micro expression training program to use videos instead of static images. The higher ecological validity of this approach is reflected in the large effect size of the improvement in micro expression processing ability of the participants. In summary, this exploratory study shows that micro expression training has promise in enhancing recognition skills and mental health, meriting further exploration through larger, controlled trials.

In conclusion, the current investigation has demonstrated that training in micro expressions may yield beneficial effects in terms of proficiency in this skill and mental health. Future studies can be conducted in order to further explore the magnitude and durability of these findings more comprehensively, employing an expanded cohort and a methodologically rigorous, randomized controlled trial framework.

## Methods

This study was pre-registered on OSF (https://osf.io/tv9xr/).

### Participants and recruitment

The study sample consisted of 28 participants, recruited through the Prolific platform. They were reimbursed £2.50 for the initial testing, £2.50 for each of 4 training sessions (described in the procedure section), and £2.50 for the final testing. They received an extra £2 bonus for completing the whole study. Thus, in total, participants were reimbursed for up to £17. We recruited participants who had reported depression symptoms in Prolific’s pre-screening questionnaires. Due to the nature of Prolific pre-screening, the answers are given a substantial amount of time prior to the time of testing.

#### Inclusion and exclusion criteria

Participants inclusion criteria included being aged 18 and over, and a native speaker or equivalently fluent in English. The only exclusion criterion was the non-usage of antidepressant drugs, as they have been demonstrated to affect facial expression recognition^29^ and would affect the participants’ BDI scores. Since the Prolific platform does not provide the capability to exclude participants based on antidepressant usage beforehand, they had to be asked about it at the beginning of the study. None of the participants who attempted taking part in the study used antidepressants, and thus none had to be removed for this reason.

#### Sample size determination

Previous research has indicated large effect sizes of micro expression training, such as η^2^ = 0.21^19^ and ε^2^ = 0.23^20^. Using G*Power 3.1.9.7, with a Cohen’s *f* of 0.58 (equivalent to η^2^ of 0.21) and power of 0.95, it was determined that a sample of 12 would be necessary to capture an effect of that size. Due to the recruitment of a low mood sample, additional measurements also being used, as well as possible attrition, we aimed for a sample size of 30. Participants were informed that they were able to withdraw from the study at any time by leaving the study webpage, which resulted in them not receiving a reimbursement for that part of the study.

### Design

This study used an open-label, single-group longitudinal design. There was no control group, and the participants were aware of what they were being trained in. The primary independent variable was the time of testing (pre training/post training). The dependent variables for the three hypotheses were micro expression recognition, depressive symptoms, and social functioning, respectively. Demographic variables (age, gender, education) were collected and used for the adjustment of the statistical analyses. The study was completed online.

### Measures and materials

#### Micro expressions training videos

The METV^30^ is based on the Facial Action Coding System rules developed by Paul Ekman and Wallace V. Freisen^31^. However, contrary to other micro-expression recognition programs such as Paul Ekman’s Micro Expression Training Tool^26^, the METV program does not use pictures of faces but instead uses videos showing facial expressions in a real interaction. The stimuli showed videos with a Caucasian face of a male or female, looking straight to the camera, showing only one variation of micro expression in normal speed for each video. The duration of the micro expressions was 0.5 s or shorter. Micro expressions shown on the faces were triggered naturally and comply with the Facial Action Coding System coding rules^31^. All videos included in the METV, both for training and research purposes, were performed by actors who consented to these videos being used for all purposes. They were coded by two independent certified Facial Action Coding System coders, guaranteeing compliance with the rules. The selection of the videos for the METV Research test was based on the theory of facial muscle movements. Some micro expressions, such as anger or sadness, can be shown on the face in a number of different ways. Surprise and fear have fewer variations, disgust and contempt even fewer, and happiness is primarily determined by the position of the lips^32,33^. The METV research test shows one video for each muscle movement indicative of an emotion. Therefore, it consists of 20 videos: 4 micro expressions of anger, 4 of sadness, 3 of fear, 3 of surprise, 2 of disgust, 2 of contempt, 1 of happiness, and 1 neutral face). The METV research test takes about 10 min. The METV test is a forced choice test of one from eight possible answers (representing each emotional category that is displayed across the test), given after each of the stimuli is presented. The METV test allows to answer up to 3 times, if the initial answer is not correct. After three wrong answers, the participant is presented with the correct answer. In the present study, the score was calculated as the percentage of correct answers given on the first attempt.

#### The beck depression inventory-II

The Beck Depression Inventory-II was used to measure depressive symptoms. It consists of 21 items asking the participant to describe themselves for the past two weeks, on how much did they experience certain depressive phenomena, such as agitation or concentration difficulty^34^. Items are answered on a 4-point Likert-type scale ranging from 0 to 3. Due to recommendations from the ethical board, item Q9 (suicidal ideation) was removed. Scores are calculated by adding up all the answers (range 0–60), and higher scores indicate more severe depression. The scale has excellent reliability (α = 0.90) and validity^35^. In the present study, it also showed excellent reliability (α > 0.90).

#### Social functioning questionnaire

The Social Functioning Questionnaire was used for the measurement of social functioning^36^. It consists of 8 items regarding the participants’ functioning in the past two weeks. The items are answered on a 4-point Likert-type scale ranging from 0 to 3. The total score, which is calculated by adding up the answers, can range from 0 to 24. Higher scores indicate worse social functioning. It was created after the Social Functioning Schedule, which has good psychometric properties^37^. In the present study, based on Cohen’s criteria^38^, it showed acceptable reliability (Cronbach’s α = 0.685–0.744).

#### Sociodemographic data

Sociodemographic factors including age in years, gender (male, female, other), and highest level of education were also measured. There was no explicit definition of gender used in the study, due to it only being used to adjust analyses, and not as a primary research variable.

### Procedure

Ethics approval was obtained from the Faculty of Science Research Ethics Committee at the University of Bristol (Approval Code: 2024-17933-20,020). The study was conducted according to the revised Declaration of Helsinki (2013) and the 1996 ICH Guidelines for Good Clinical Practice E6(R2). Data collection started on 15/03/2024, and ended on 10/06/2024. The investigator explained the nature, purpose and risks of the study to the participants in an online information sheet before they consented to participate in the study by clicking a button. Participants were informed that they were free to withdraw at any time by simply closing the web page. Thus, all participants were provided informed consent during the study. All data was anonymized before analysis.

**Fig. 7:**
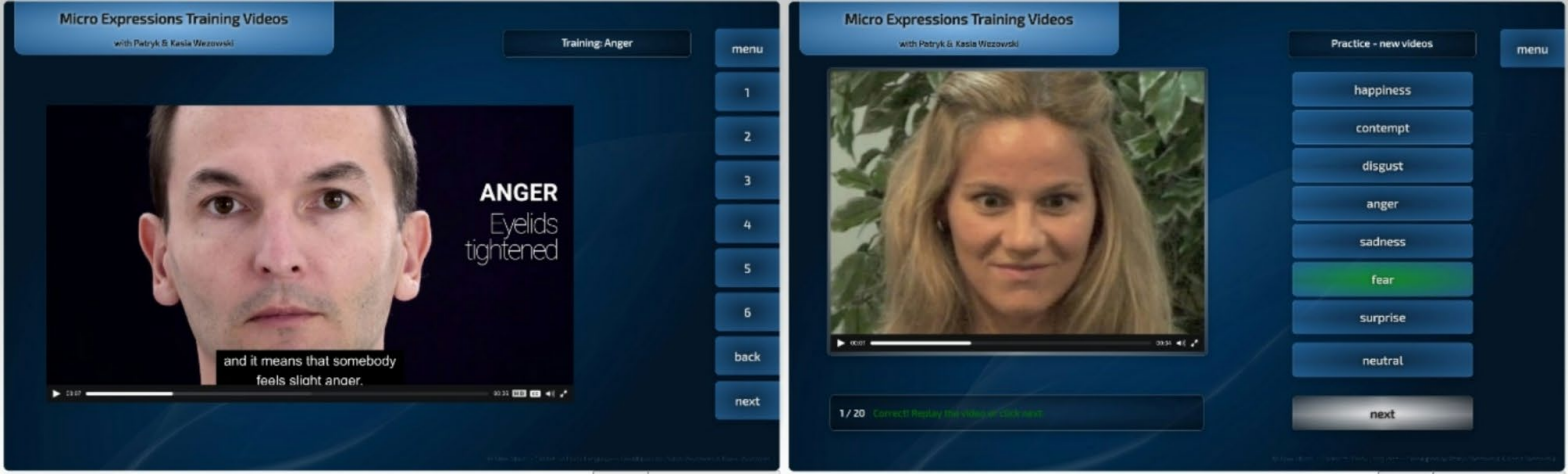
Screenshots from the micro expression training videos. Image on left represents a screenshot of the instructional videos, while the image on the right represents a screenshot of the training videos. Faces shown in videos are of actors who gave consent for the usage of these videos for all purposes.

**Fig. 8:**
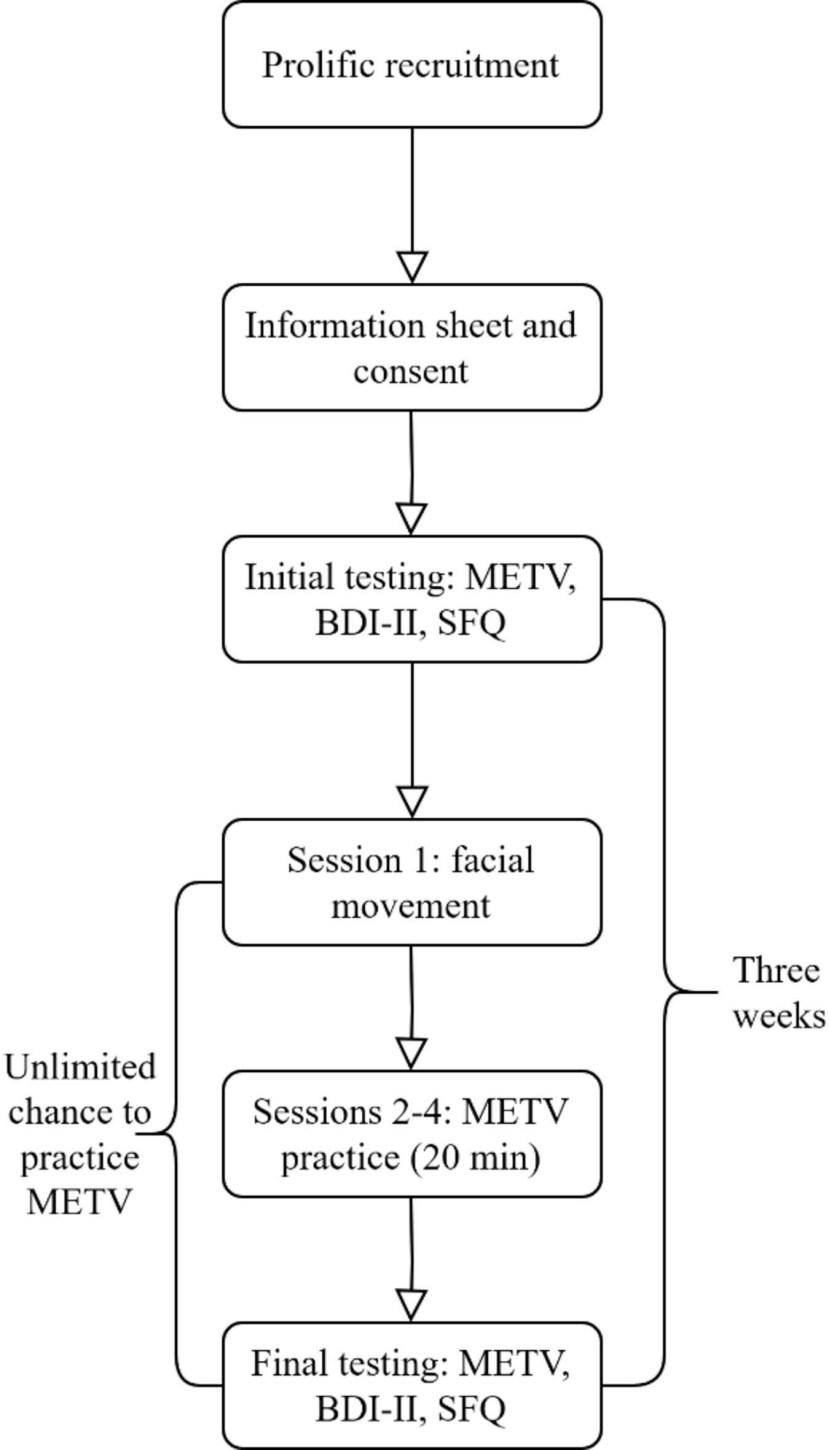
Flowchart of the study procedure.

The participants were tested at two time points: at the start of taking part in the study and after three weeks. At both time points, they completed the METV, the BDI-II, and the SFQ. Between the two time points, they trained micro expression recognition. This began with one session of participants learning about theory regarding micro expression recognition. They were demonstrated the 28 ways in which facial muscles can move, indicating different emotions (example from the course in Fig. 7). The videos helped participants become acquainted with micro expressions and learn how to recognize them. These videos were presented in slow motion, so that the participants could get a precise grasp of the muscle movements. Afterwards, in order to fully ingrain the knowledge and perception of micro expressions, they had three sessions of training with METV videos different from the ones used for the test. In the training sessions, they were demonstrated videos of micro expressions (which are up to 0.5 s in length), and had to answer which micro expression was presented (Fig. 7). They were allowed to make infinite attempts, and were given feedback on whether the answer was correct or incorrect. Each of the sessions took around 20 min. They received reminders to do these activities. They were also able to log in and practice METV any number of times they liked, outside of the three sessions. The full procedure may also be observed in Fig. 8. In total, the procedure was expected to teach participants both the theory of micro expressions and how to recognize them.

### Statistical plan

#### Data screening

There were two data screening criteria used in the present study. First, extreme outliers—defined as those participants whose METV, BDI-II, or SFQ test scores lie more than 3 times the interquartile range below the first quartile or above the third quartile—were removed from the dataset. Less extreme outliers—defined as those participants whose METV, BDI-II, or SFQ scores lie between 1.5 and 3 times the interquartile range below the first quartile or above the third quartile—were included in primary analyses but their influence was investigated through sensitivity analyses that exclude them. Participants that did not comply with the basic instructions of the study (e.g., used Chrome browsers or smart phones, which are not compatible with the METV), were removed from the dataset, without replacement. Further, for all statistical analyses conducted for this study, all relevant assumptions (such as normality of data and homoscedasticity of the error terms) were verified prior to analysis. Should any data not fulfil the requirements for the analysis in question, appropriate mitigation measures were undertaken.

#### Analysis plan

The data was analysed using a series of repeated-measures analyses of covariance (ANCOVAs). This analysis differs from the one indicated in the study protocol on OSF due to the change in design from having two groups to only one, which was done due to budget constraints. In all models, the within-groups factor was defined by the two measurements of the dependent variable. The dependent variables were the scores on METV (Hypothesis 1), BDI-II (Hypothesis 2), and SFQ (Hypothesis 3). All analyses were presented both unadjusted and adjusted for demographics. Data was analysed using SPSS v28. Dataset and analysis syntax are available on OSF^39^.

## Data Availability

Study data and code used for analysis is available on OSF: https://osf.io/tv9xr/. Screenshots in Fig. 7 are in the appendix to the PhD thesis of Kasia Wezowski, which is accessible for all researchers at the university of Bristol. Otherwise, these micro expression videos are restricted only to people who receive a login to use the METV program. All videos included in the METV, both for training and research purposes, were performed by actors who consented to these videos being used for all purposes. This includes the screenshots presented in Fig. 7.
